# To be or not to be aphasic: use of story retelling as a marker in subclinical aphasia

**DOI:** 10.3389/fnhum.2025.1701696

**Published:** 2026-01-13

**Authors:** Jacquie Kurland, Anna Liu, Polly Stokes

**Affiliations:** 1 Department of Speech, Language, & Hearing Sciences, University of Massachusetts Amherst, Amherst, MA, United States; 2 Department of Mathematics and Statistics, University of Massachusetts Amherst, Amherst, MA, United States

**Keywords:** aphasia, subclinical aphasia, story retelling, main concepts, communication success

## Abstract

**Purpose:**

This study examined story retelling in individuals with aphasia who scored at or above the 93.8 cutoff on the Aphasia Quotient (AQ) of the Western Aphasia Battery-Revised (WAB-R). The performance of these participants deemed “not aphasic by WAB” (NABW) was compared with the performance of non-aphasic participants and individuals with anomic aphasia.

**Method:**

Most participants were from a test development dataset for the Brief Assessment of Transactional Success in communication in aphasia (BATS), including four groups of 16 individuals: (1) a group who tested NABW; (2) a group with anomic aphasia matched on gender, age, education, and time post-onset; (3) a group with mild anomic aphasia who scored just below the NABW cutoff; and (4) a group of non-aphasic individuals matched on gender, age, and education with the NABW group. Groups were compared on main concepts of the BATS story retelling. Groups with aphasia were also compared on the main concepts of stories retold by non-aphasic conversation partners following co-construction of stories and on self-reported scores of the impact of aphasia on everyday communication.

**Results:**

The results showed significant differences in the retelling of the story’s main concepts between the non-aphasic control and conversation partner groups, with non-monotonic decreases in performance in comparisons of groups with and without aphasia: from non-aphasic to NABW to mildly anomic to anomic. Individuals deemed NABW (and their conversation partners) did not perform significantly better than individuals with mild anomic aphasia (and their conversation partners) on story retell main concepts. There were significant differences in the production of AphasiaBank discourse main concepts between the group with anomia and both the non-aphasic and NABW groups, but not between the non-aphasic and NABW or those with mild aphasia.

**Conclusion:**

Individuals with aphasia who scored “non-aphasic” on the WAB demonstrated impairments in story retelling that align with their self-report of diminished everyday communicative functioning. This finding adds to growing support for the addition of a new measure of functional communication to the core outcome set of measures utilized in aphasia research. We propose the BATS, a measure that is sensitive across the spectrum of aphasia severity, including cases of mild and subclinical aphasia.

## Introduction

Over the last decade, clinical researchers in aphasiology have begun to describe a new subpopulation of individuals whose complaints align with a diagnosis of aphasia but whose test scores on standardized tests suggest their language is unimpaired. They have been referred to as having very mild aphasia (Cavanaugh and Haley, 2020), more often as having latent aphasia (DeDe and Salis, 2020; Silkes et al., 2021), and at times described as subclinical, non-clinical, or “not aphasic by WAB” (NABW; Fromm et al., 2013; as cited in Fromm et al., 2017). Although it is unsatisfying to describe a group that self-identifies as having aphasia by the absence of clinical pathology, NABW is an apt term and a reminder that our outcome measures are not always sensitive to the full range of presentations of aphasia. Given that Pichot (1955), who first coined the term “latent aphasia,” referred to a decline in vocabulary among patients with arteriosclerotic dementia, going forward, the terms NABW or subclinical aphasia will be used in reference to the group under investigation in the current study.

Individuals with mild aphasia can experience devastating consequences to their everyday language use despite being judged by language outcome measurement instruments such as the WAB as “normal” or “only mildly” impaired (Armstrong et al., 2013). For example, in semi-structured interviews with five individuals with “very mild aphasia,” all participants described being challenged in every aspect of communicative life participation (Cavanaugh and Haley, 2020). Beyond the potentially damaging invalidation of this group’s lived experience, the danger of this misclassification is the denial of services. The Western Aphasia Battery-Revised (WAB-R; Kertesz, 2007) has been identified as the recommended outcome measurement for language reached by international consensus (ROMA; Wallace et al., 2019). Yet Kertesz (2007) recommends that a WAB Aphasia Quotient (WAB-AQ) at or above 93.8 suggests that “the patient may be considered normal or non-aphasic” (p. 91). In effect, the WAB has created a new aphasia classification for individuals with mild anomic aphasia who score above the WAB-AQ cutoff yet experience persistent communication disability.

Although these individuals score within normal limits on the WAB, there are many reasons to suggest that the WAB-AQ cutoff decreases the test’s sensitivity, producing too many false negatives. In the literature examining discourse production in this group, it has been demonstrated that participants deemed NABW produce connected speech that is more disfluent (Fromm et al., 2024), contains fewer core lexical items and main concepts (Dalton and Richardson, 2015; Fromm et al., 2017; Stark et al., 2024), contains vocabulary that is less lexically diverse and has more errors (Fromm et al., 2017), and differs in temporal measures including speech rate and silent pause duration, especially tied to the introduction of new episodes in narrative storytelling (DeDe and Salis, 2020), when compared to non-aphasic control speakers.

Most studies examining differences in narrative production between individuals deemed NABW and non-aphasic controls have used discourse data from the AphasiaBank (MacWhinney et al., 2011), often using the Cinderella story retell, which, at last count (Fromm et al., 2024), included 257 controls and 281 people with aphasia, including 31 who scored NABW. By far the largest database, the AphasiaBank has made tremendous contributions to clinical research in aphasia. This includes identifying aspects of monologic narrative production in individuals classified as NABW that differ from those of controls, as indicated above. Thanks to the AphasiaBank, aphasiologists are much better poised to perform cross-study analyses in alignment with the goals of the ROMA project.

Although the rich and growing literature on differences between subclinical and non-aphasic monologic discourse validates the lived experience of individuals with subclinical aphasia, monologic picture descriptions and story retells are insufficient to serve as a communication outcome measure in aphasia. The consortium currently developing a core outcome set (COS) for improving Research Outcome Measurement in Aphasia (ROMA; Wallace et al., 2019; ROMA-2; Wallace et al., 2023) recently identified The Scenario Test (TST; van der Meulen et al., 2010) as a core outcome measurement instrument for communication (ROMA-2; Wallace et al., 2023). As the name suggests, TST sets up role-playing scenarios in an interactive setting to assess multimodal communication. Theoretically grounded in the ‘situated language use’ model (Clark, 1996; Doedens and Meteyard, 2018), TST embodies the model’s fundamental principles, i.e., that real-world communication is defined by three core characteristics: (1) interactivity, (2) multimodality, and (3) context-sensitivity. During role-playing scenarios, a person with aphasia and a dyadic partner can use verbal or nonverbal means of communication to co-construct meaning, giving TST strong face validity. While TST is an excellent choice for individuals with moderate to severe aphasia, it is prone to ceiling effects for individuals with mild to moderate aphasia (Doedens and Meteyard, 2020). Thus, the ROMA-2 consortium has advised that “… consideration of the optimal way to measure communication with people with mild language impairments is needed” (p. 1026).

A new tool that could fill this gap is the Brief Assessment of Transactional Success in communication in aphasia (BATS; Kurland et al., 2021, 2024, 2025). Like TST, the BATS is grounded in the situated language use model but uses story retelling, rather than role-playing, as the vehicle for assessing real-world communication. Like earlier story retelling paradigms using I Love Lucy episodes (Ramsberger and Rende, 2002) and Mr. Bean episodes (Carragher et al., 2023), the BATS utilizes co-construction of meaning to assess communication in aphasia. Stories are watched and/or listened to by an individual with aphasia who must rely on audiovisual memory to first retell the story to the test administrator. Next, the BATS story is retold to a non-aphasic conversation partner who is unfamiliar with it to reach common ground on the story gist and details. Finally, following a six-minute topic-constrained conversation in which the story is co-constructed, ecologically valid evidence of communication success can be obtained. This is done by assessing the presence, accuracy, and completeness of main concepts (Richardson and Dalton, 2016, 2020), or story gist, in the partner’s story retell, which is a product of the dyad’s co-construction of meaning during their conversation.

The use of co-constructed communication tasks such as story retelling has several advantages over other discourse tasks such as dyadic unconstrained conversations. Eliciting, transcribing, and analyzing monologic aphasic discourse samples are prohibitively labor-intensive and pose a major obstacle to their widespread use as an outcome measure by clinicians (Bryant et al., 2017; Cruice et al., 2020). Using natural conversation would be exponentially more impractical. Without having external criteria, such as an original story, with which to compare transactional success in conversation (Ramsberger and Rende, 2002), the task of measuring communication success would be out of reach for most clinicians. The BATS, which is in development to automate the processes of story retell collection, transcription, and main concept scoring (Kurland et al., 2025), could eventually be a clinically feasible communication outcome measure in aphasia, including in subclinical aphasia.

One reason why the BATS tool may be more sensitive to communication disability in people with subclinical aphasia is that retelling a story without the benefit of having a picture to refer to is, by design, more challenging than most of the spontaneous speech, auditory verbal comprehension, repetition, and naming and word-finding tasks that comprise scores in the WAB-AQ. The BATS task taps into complex verbal and nonverbal processing, including perception and integration of auditory and visual information, and storage and recall of story gist and details. During co-construction of the story with a partner who is unfamiliar with the story, multiple nonlinguistic processes, including attention, working memory, and executive functions, support an individual’s ability to accurately and completely convey the story while monitoring one’s own speech as well as the conversation partner’s verbal and nonverbal cues regarding how well they are understanding the story. The task taps into the complex web of interwoven psychological mechanisms and linguistic processes that reflect real-world communication. Thus, it is not coincidental that among the explanations advanced as plausible contributing factors underlying functional language impairment in mild and so-called latent aphasia are attention (Murray et al., 1998, 2006), verbal short-term and working memory (Salis et al., 2021; Silkes et al., 2021), executive functions (Frankel et al., 2007), and temporal aspects of word processing (DeDe and Salis, 2020; Salis et al., 2021; Silkes et al., 2021).

This manuscript addresses a gap in current methods of assessing communication outcomes in very mild and subclinical aphasia. We propose a story retelling tool that is sensitive across aphasia severity, unlike measures of language and communication currently in a core outcome set for aphasia. In the current study, we aim to demonstrate the tool’s sensitivity to subclinical and mild aphasia by comparing story retells from individuals who scored NABW to those of individuals with anomic aphasia, mild anomic aphasia, and non-aphasic controls. The study is a post-hoc analysis of a subset of the BATS dataset, which we further analyzed to examine the phenomenon of subclinical aphasia in the context of an everyday communicative task: retelling a story to someone who is not already familiar with it. Of the 96 individuals with aphasia who participated in the most recent phase of BATS test development (Kurland et al., 2025), 16 scored NABW but self-identified as having aphasia. They described signs and symptoms such as the inability to return to work due to impaired ability to keep pace with the communication demands, a trait that they attributed to post-stroke aphasia. This is consistent with previous small studies of individuals with mild aphasia (Raymer and LaPointe, 1986; Cavanaugh and Haley, 2020).

Given the disparity between their language performance on the WAB and their self-perceived communication disability, the current study aimed to address this difference by examining whether individuals who are scored “non-aphasic” nonetheless demonstrate impairments in story retelling that align with their self-report of diminished everyday communicative functioning. Groups along a continuum of anomic severity were compared to each other and to actual non-aphasic participants in both story retelling and traditional monologic discourse tasks. Furthermore, their conversation partners’ story retellings, following the co-construction of the stories, were compared.

The performance of 16 individuals deemed NABW in producing main concepts in BATS story retells, as well as the performance of their 32 (familiar and unfamiliar) conversation partners, was compared with that of three other groups: (1) a group with anomic aphasia who were matched on gender, age, education, and time post-onset and their conversation partners; (2) a group with mild anomic aphasia who scored just below the NABW cutoff (mild anomic) and their conversation partners; and (3) a group of non-aphasic individuals matched on gender, age, and education with the NABW group who participated in an earlier phase of test development (Kurland et al., 2021). The three groups with aphasia are compared in two additional ways: (1) in their self-assessment of how effectively they perform in a variety of communication scenarios using the Aphasia Communication Outcome Measure (ACOM; Hula et al., 2015); and (2) with a non-aphasic group matched on gender and race/ethnicity in production of main concepts using traditional monologic AphasiaBank discourse stimuli.

In alignment with participants’ self-perceptions, we expected the BATS story retelling tool to be more sensitive to communication disability in individuals classified as NABW than either WAB-AQ scores or performance on traditional discourse tasks would suggest. We hypothesized that, on average, main concept scores for story retelling across the four groups would decline from non-aphasic controls to NABW to mild anomic to anomic. We expected higher scores but with similar patterns in main concept scores between groups for traditional picture descriptions. We also hypothesized that self-reported scores of the impact of aphasia on everyday communication would not differ significantly between the NABW and mildly anomic groups, reflecting both groups’ self-perception of communication disability.

## Materials and methods

### Participants

Eight groups of participants were compared on two tasks. Three groups of participants with aphasia (subclinical, mild, and anomic) were compared with non-aphasic participants on the BATS story retelling task. Furthermore, following topic-constrained conversations in which the stories were co-constructed, three groups of conversation partners were compared with non-aphasic participants on story retells. To compare story retelling with the more traditional discourse elicitation tasks, the same three groups of participants with aphasia were compared with a non-aphasic cohort from the AphasiaBank on monologic narratives. In total, 48 persons with aphasia (PWA), 48 familiar conversation partners (FCP), and 48 unfamiliar conversation partners (UCP) comprised three groups of PWA/CP story retell dyads: (1) NABW (*n* = 16; WAB AQ range: 93.9–98); (2) matched anomic (*n* = 16; WAB AQ range: 78.6–88.6); and (3) mild anomic (*n* = 16; WAB AQ range: 89.1–93.4). The matched anomic group was matched with the NABW group on sex, race/ethnicity, age, education, and time post-onset. The mild anomic group, also referred to as “Top Anomic,” was comprised of the next-highest-scoring group of 16 participants with anomic aphasia who scored below the WAB AQ cutoff of 93.8. All three groups were fairly well matched on the demographic parameters listed above (see Table 1). One group of non-aphasic controls (NC; *n* = 16) who participated in an earlier phase of BATS tool development (Kurland et al., 2021) was matched with the NABW group on gender, age, race/ethnicity, and education. A second group of NC (*n* = 16) selected from the AphasiaBank for comparison to PWA groups’ picture description monologues was matched on gender and race/ethnicity. Clinical and demographic group information on all participants is shown in Table 1.

**Table 1:** Clinical and demographic characteristics for aphasic and non-aphasic groups: means and standard deviations (*sd*).

Parameter (participants with aphasia)	Anomic (*n* = 16)	Mild (“Top Anomic”) (*n* = 16)	NABW (*n* = 16)
Sex (% female)	62.5%	62.5%	62.5%
Race/ethnicity (% Caucasian)	75.0%	81.3%	81.3%
Age in years (*sd*)	62.4 (*9.9*)	59.1 (*7.5*)	60.6 (*9.1*)
Education in years (*sd*)	16.7 (*2.2*)	16.8 (*3.3*)	16.9 (*2.5*)
Time post-onset in years (*sd*)	6.2 (*5.8*)	6.2 (*4.6*)	5.9 (*6.0*)
WAB-R AQ	86.1 (*2.9*)	91.6 (*1.4*)	95.8 (*1.3*)
WAB-R AQ (range)	78.6–88.6	89.1–93.4	93.9–98

Parameter (non-aphasic participants)	CP paired w/anomic (*n* = 32)	CP paired w/mild anomic (*n* = 32)	CP paired w/NABW (*n* = 32)	Non-aphasic controls (BATS story retells; *n* = 16)	Non-aphasic controls (AphasiaBank monologues; *n* = 16)
Sex (% female)	71.9%	71.9%	75.0%	62.5%	62.5%
Race/ethnicity (% Caucasian)	90.6%	81.3%	78.1%	81.3%	81.3%
Age in years (*sd*)	48.3 (*20.5*)	50.0 (*17.8*)	48.6 (*20.3*)	60.3 (8.2)	29.0 (*7.1*)
Education in years (*sd*)	16.1 (*3.0*)	18.1 (*2.8*)	16.0 (*3.3*)	17.0 (2.7)	15.8 (*2.6*)
TICS (CP) or MMSE score (NC)	36.0 (*1.1*)	36.2 (*1.8*)	36.3 (*1.7*)	29.7 (*0.7*)	DNT

NABW, not aphasic by WAB; WAB-R, Western Aphasia Battery-Revised (Kertesz, 2007); AQ, Aphasia Quotient; CP, conversation partners; TICS, Telephone Interview for Cognitive Status (Brandt et al., 1988); MMSE, Mini-Mental’ State Exam (Folstein et al., 1975); DNT, did not test.

Inclusion criteria for all BATS story retell groups were 18 years or older, fluent in English, with normal or corrected vision and hearing, no history of neurological conditions other than left hemisphere stroke in the aphasia group (at least 3 months post-onset), medically stable, willing to be videotaped retelling stories, and able to participate in study sessions via Zoom. Exclusionary criteria included a history of significant psychiatric disease, drug or alcohol dependency, TBI with loss of consciousness and/or significant cognitive sequelae, chronic medical conditions likely to impair cognition, presence of visual field cuts or visual neglect, lack of technical skill or other resources for participating via Zoom. Screens were administered by telephone or over Zoom during the initial screening and consenting process. Participants with aphasia were screened using the Auditory Verbal Comprehension subtest of the Western Aphasia Battery (WAB-R; Kertesz, 2007), with a minimum required score used to calculate the aphasia quotient of 4.0. No cognitive screening was performed. The Telephone Interview for Cognitive Status (TICS; Brandt et al., 1988) was used as a cognitive screen for non-aphasic conversation partners. CP TICS scores (mean = 36.1; sd = 1.6; range = 31–41) were all within normal limits according to Brandt et al. (1988), in which “normal” participants scored between 31 and 39. The Mini-Mental State Exam (MMSE; Folstein et al., 1975) was used for the same purpose on the non-aphasic control group in an earlier phase of in-person BATS test development. CP scores on the MMSE (mean = 29.7; sd = 0.7; range = 28–30) were also within normal limits according to Folstein et al. (1975), where scores of 25–30 indicate “normal cognitive function.” No cognitive screening information was available for the AphasiaBank group of 16 matched non-aphasic controls who provided monologic picture descriptions for comparison to the groups with aphasia.

The institutional review board of the University of Massachusetts Amherst approved both studies. Informed consent was obtained from non-aphasic control subjects in person and from all other participants via phone or video conferencing software, with signatures obtained via DocuSign.

### BATS story retell data acquisition and main concept analysis

The BATS library of stimuli currently consists of 16 short video and audio clips previously described in detail. Similarly, methods of data acquisition, transcription, and main concept analyses have been described in detail for non-aphasic controls (Kurland et al., 2021) and for participants with aphasia and their non-aphasic conversation partners (Kurland et al., 2024, 2025). The following is an abbreviated summary of these methods.

The library of stimuli included 16 short (2–3 min) video and audio stimuli in four stimulus types that varied along a continuum of dependence on auditory comprehension for full understanding of the story gist. These included (1) non-verbal short stories that were either humorous or about “doing good”; (2) how-to videos about home improvements wherein the verbal and visual messages were tightly aligned; (3) short autobiographical stories from the PBS “Brief but Spectacular” series in which there was visual support for the story being narrated; and (4) speech-dependent audio clips from the NPR “StoryCorps” series that included a single still photo for visual support.

In the first phase of the BATS tool development, non-aphasic controls each retold eight stories in person, including two from each of the four stimulus types, in one 1-h testing session. After watching or listening to each stimulus, they were instructed to “retell what the story was about in as much detail as you can remember.” This resulted in 128 story retell samples from the subset of 16 non-aphasic controls. Their data were part of a larger set of story-retelling normative references from which checklists of main concepts for each BATS stimulus were developed (Kurland et al., 2021).

In a more recent phase of tool development, participants with aphasia were paired with familiar and unfamiliar conversation partners in two separate one-hour testing sessions conducted over 1–2 weeks. The order of sessions was counterbalanced for conversation partner familiarity. In each session, participants with aphasia each viewed and retold four stories over Zoom, including one from each of the four stimulus types. Like the normative sample, after watching or listening to each BATS stimulus, they were instructed to “retell what each clip was about, in as much detail as you can remember.” Immediately after each story retell, depending on the session, the familiar or unfamiliar partner was brought from a Zoom waiting room. Participants were instructed to engage in a six-minute conversation to reach a shared understanding of what the clip was about so that the conversation partner could then retell the story in as much detail as possible. They were encouraged to use any verbal or nonverbal modality (gesture, writing, drawing, etc.) as needed to reach common ground on what the story was about. After 6 min, the conversation partner was asked to retell the story, using the same instructions. The participant with aphasia was asked not to comment during the partner’s story retell. The test administrator introduced dyads as needed for unfamiliar partners, gave instructions, presented the stimuli, and kept time during the conversations, but did not participate in any way in the story retell processes. She was visible to all participants except during the presentation of the stimuli. This phase resulted in a total of 768 story retell samples from the three groups of individuals with aphasia investigated in the current study. Transcripts were generated from de-identified audio files using Assembly AI’s speech-to-text AI models.^1^ Reliability of transcript accuracy was very high, as described in Kurland et al. (2024, 2025).

For the current post-hoc study of a subset of the BATS dataset, we used scores previously obtained through an adapted version of the semi-automated open-source web-app, mainConcept (Cavanaugh et al., 2021, 2022), to measure presence, accuracy, and completeness of main concepts (MC). An MC composite ratio was used to enable comparisons across BATS stimuli, which differed in the number of main concepts (Kurland et al., 2021). The ratio was calculated by summing MC scores and dividing by the number of main concepts for each stimulus, resulting in a score between 0 and 1. For each story reteller with aphasia, or each combined familiar and unfamiliar conversation partner of each participant with aphasia, a mean MC composite (MCComp) score was calculated over the eight story retells.

### Traditional monologues data acquisition and main concept analysis

Three stimuli, including two picture series (Broken Window and Refused Umbrella) and one procedural discourse task (Sandwich) from the AphasiaBank protocol (MacWhinney et al., 2011), were presented to participants with aphasia via Zoom during a testing session before the BATS story retell sessions. As with methods previously described for story retells, transcripts for de-identified Zoom audio recordings were generated using Assembly AI. Transcripts were scored for presence, accuracy, and completeness of main concepts using an adapted version of Cavanaugh et al.’s (2021, 2022) web-app, mainConcept.

Transcripts from the same three AphasiaBank stimuli were acquired from 16 participants in the Richardson library, whose data acquisition methods are described in Richardson and Dalton (2016, 2020). Because the sample is much younger than the BATS sample, we could not match the AphasiaBank non-aphasic control group with our NABW group on age or education, but we selected participants to most closely match them on gender, race/ethnicity, age, and education.

A research assistant was trained to use the mainConcept app on a different set of AphasiaBank transcripts until she reached 100% inter- and intra-rater reliability with the first author before scoring the 16 participants’ transcripts. For the current study, approximately 20% of all MC-scoring transcripts were interpreted by the first author, blinded to each other’s results. Inter-rater agreement on the monologue MCs was 94.4%. Disagreements in MC summary scores greater than 3 points were resolved by the first author. As with story retell data, we calculated mean MCComp ratio scores for each participant.

### Self-report of communication effectiveness data acquisition

The adaptive version of the online open-source web-app, Aphasia Communication Outcome Measure (ACOM; Hula et al., 2015), was used to collect participants’ self-reported perceptions of their communication effectiveness. The ACOM was chosen over other patient-report measures due to its demonstrated psychometric robustness and its online administration. Twenty-one items in five content areas (talking, comprehending, writing, naming, and general factors) were acquired from each participant with aphasia in a remote test session before the BATS story retell test sessions. The instructions were presented via the shared screen function in Zoom and read aloud by a speech-language pathologist (Stokes), using cueing and repetition for confirmation as needed, in accordance with the protocol (Hula and Doyle, 2021). Unless they could read aloud the response labels, participants with aphasia were trained to use the Zoom marker tool to enable remote ‘pointing’ to the scale for rating, “*How effectively do you …*?” Although a 12-item adaptive version was available, it was recommended that we acquire at least 20 of the 59 items in the bank when using the adaptive ACOM (W. Hula, personal communication, July 5, 2022).

### Statistical analysis of BATS and monologues data

We fit a one-way ANOVA model with four levels of the WAB-AQ grouping variable: (1) non-aphasic controls; (2) subclinical aphasia (NABW); (3) mild anomia; and (4) anomia for both conversation partner/non-aphasic control comparison groups and participant with aphasia/non-aphasic control comparison groups. Although the WAB-AQ factor includes only the three groups of participants with aphasia who were tested on the WAB, the non-aphasic control group (NC) was included for comparison purposes. Given that NCs do not have conversation partners, we used NCs’ scores as their “CP scores.” We also performed pairwise comparisons among the four levels, using Sidak’s adjustment for multiple tests.

### Descriptive analysis of ACOM data

We performed a post-hoc descriptive analysis of items that most respondents answered and that were most closely associated with the story retell task, including five items: (*How effectively do you…*) (1) … *talk to your closest family member or friend?* (2) … *keep a conversation going?* (3) … *tell a joke?* (4) … *tell a story?* and (5) … *have a conversation with strangers?*

## Results

### BATS group comparisons

**Figure 1:**
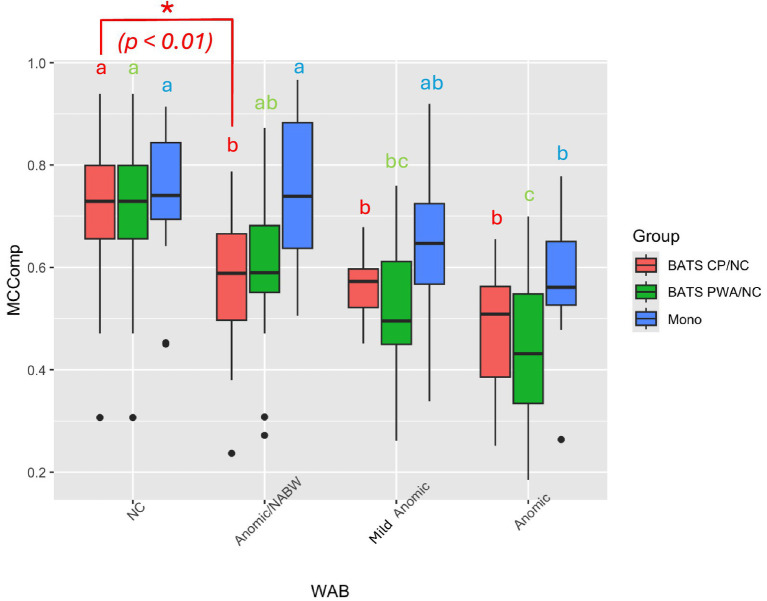
Comparisons of main concept composite scores within each group among non-aphasic (NC), not aphasic by WAB (NABW), mild anomic, and anomic aphasia classifications. Groups include conversation partners retelling stories (BATS CP/NC; red), people with aphasia (PWA) or NCs retelling stories (BATS PWA/NC; green), and PWA or NC monologues describing pictures or procedural discourse (mono; blue). For the NCs, the BATS PWA retells are the same as the BATS CP retells.

The one-way ANOVA model discriminated between BATS CP and NC story retells (red bars in Figure 1), as well as BATS PWA and NC story retells (green bars in Figure 1), as the clear descending trend within each color group demonstrated. Specifically, on average, from Table 2A, NC produced 71% of the main concepts in the stories, followed by 57% produced by Anomic/NABW CPs, followed by 56% produced by Mild (“Top Anomic”) CPs and 48% produced by Anomic CPs, all with a standard error of 0.03. From Table 2B, NC produced 71% of the main concepts in the stories, followed by 60% produced by Anomic/NABW PWAs, followed by 52% produced by Mild (“Top Anomic”) PWAs and 44% produced by Anomic PWAs, all with a standard error of 0.04. This is expected behavior because the conversation ability, as measured by Main Concept here, should decrease from NC, Anomic/NABW, Mild Anomic, to Anomic. Notably, the difference between NC and Anomic/NABW for the CP group (the first two red bars in Figure 1) is significant (*p*-value = 0.0092 from Table 2A), signifying the discriminating power of the BATS Main Concept measure. However, the Monologues (blue bars in Figure 1) did not discriminate between consecutive levels. Notably, the first two blue bars, that is, the monologue scores of NC and Anomic/NABW, are not different, with both at 74% with 0.03 standard error, and the *p*-value for the difference between them is 0.9986 (Table 3).

**Table 2:** Grouping of the four levels of WAB-R factor based on A) BATS CP/NC or B) BATS PWA/NC and contrasts between consecutive levels.

A) BATS CP/NC group	Emmean	SE	df	Lower CL	Upper CL	Group
NC	0.71	0.03	60	0.65	0.77	a
Anomic/NABW	0.57	0.03	60	0.51	0.63	b
Mild (“Top”) anomic	0.56	0.03	60	0.50	0.63	b
Anomic	0.48	0.03	60	0.41	0.54	b

Contrast	Estimate	SE	df	t. ratio	*p*-value
NC—(anomic/NABW)	0.1399	0.0454	60	3.0837	0.0092
(Anomic/NABW)—mild anomic	0.0090	0.0454	60	0.1978	0.9962
Mild anomic—anomic	0.0839	0.0454	60	1.8487	0.1942

B) BATS PWA/NC group	Emmean	SE	df	Lower CL	Upper CL	Group
NC	0.71	0.04	60	0.63	0.79	a
Anomic/NABW	0.60	0.04	60	0.52	0.67	ab
Mild anomic	0.52	0.04	60	0.45	0.60	bc
Anomic	0.44	0.04	60	0.36	0.52	c

Contrast	Estimate	SE	df	t. ratio	*p*-value
NC—(anomic/NABW)	0.1140	0.0540	60	2.1117	0.1122
(Anomic/NABW)—mild anomic	0.0743	0.0540	60	1.3751	0.4369
Mild anomic—anomic	0.0819	0.0540	60	1.5160	0.3523

WAB-R, Western Aphasia Battery-Revised (Kertesz, 2007); BATS, Brief Assessment of Transactional Success (BATS; Kurland et al., 2021); CP, conversation partners; NCs, non-aphasic control participants from Phase 1 norming study; PWA, persons with aphasia; NABW, not aphasic by WAB. Groups that share the same letters are not statistically different.

**Table 3:** Grouping of the four levels of WAB-R factor based on monologues by PWA/NC.

BATS CP/NC group	Emmean	SE	df	Lower CL	Upper CL	Group
NC	0.74	0.04	60	0.67	0.81	a
Anomic/NABW	0.74	0.04	60	0.67	0.82	a
Mild (“Top”) anomic	0.64	0.04	60	0.57	0.71	ab
Anomic	0.58	0.04	60	0.51	0.65	b

Contrast	Estimate	SE	df	t. ratio	*p*-value
NC—(Anomic/NABW)	−0.0071	0.0508	60	−0.1401	0.9986
(Anomic/NABW)—mild anomic	0.1030	0.0508	60	2.0259	0.1351
Mild anomic—anomic	0.0641	0.0508	60	1.2606	0.5113

WAB-R, Western Aphasia Battery-Revised (Kertesz, 2007); BATS, Brief Assessment of Transactional Success (BATS; Kurland et al., 2021); NCs, non-aphasic control participants from the AphasiaBank database (MacWhinney et al., 2011); PWA, persons with aphasia; NABW, not aphasic by WAB. Groups that share the same letters are not statistically different.

### Traditional monologues group comparisons

The model also discriminated between the anomic group and the NC and NABW groups, which were not significantly different from each other (Figure 1). It did not discriminate between consecutive levels, as shown in Table 3 and Figure 1.

### ACOM group comparison

With respect to ACOM scores, we examined differences between the three groups of participants with aphasia on five items that most respondents answered, and that were most closely associated with their perceptions of communication effectiveness in scenarios that, like the BATS, include conversation and storytelling. Although the small numbers of items and responses warranted only descriptive results, they do paint a picture of awareness of communication deficits that aligns more closely with BATS story retelling performance than with traditional results from standardized tests like the WAB.

**Figure 2:**
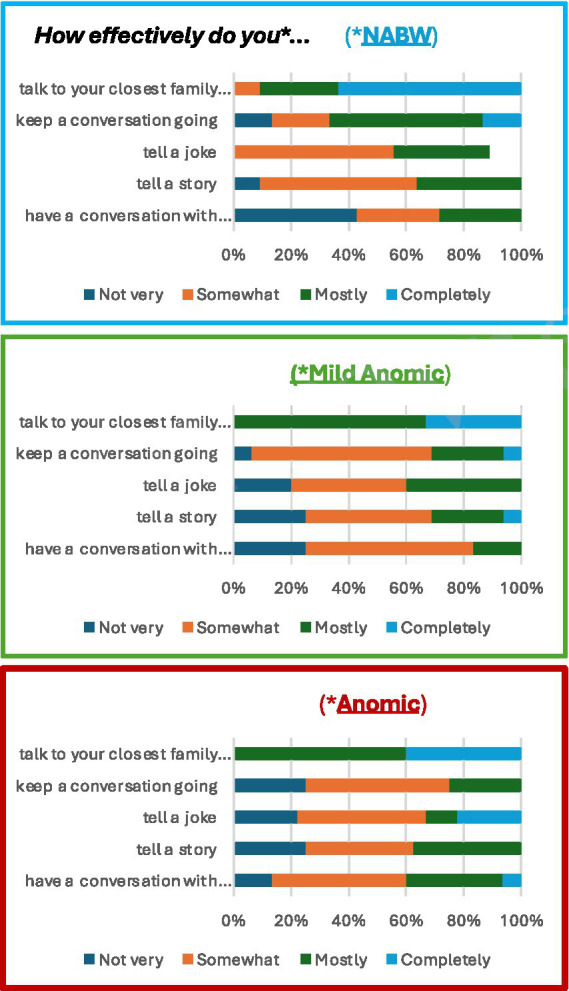
Self-ratings on five items from the Aphasia Communication Outcome Measure (ACOM; Hula et al., 2015) that were most closely aligned with aspects of story retelling with familiar and unfamiliar partners and which a majority of the 48 participants with aphasia were administered.

Of the 59 items that could have been administered by the adaptive ACOM, a majority of the 48 participants with aphasia (74%) were asked to rate their effectiveness on five items that reveal some patterns worth further scrutiny (see Table 4 and Figure 2). Two observations from the current sample highlight differences and similarities between the subclinical and the other two groups of PWA. First, unlike the Mild Anomic and Anomic groups of PWA, strong majorities of PWA deemed NABW rated themselves as “completely” effective at “talk[ing] to your closest family member or friend” (NABW: 63.6% vs. Mild Anomic: 33.3% and Anomic: 40.0%) and “mostly” or “completely” effective at “keep[ing] a conversation going” (NABW: 66.7% vs. Mild Anomic: 31.3% and Anomic: 25.0%). Second, like the Mild Anomic and Anomic PWA groups, a majority of respondents deemed NABW rated themselves as “not very” or only “somewhat” effective at “tell[ing] a joke” (NABW: 55.6% vs. Mild Anomic: 60.0% and Anomic: 66.7%), “tell[ing] a story” (NABW: 63.6% vs. Mild Anomic: 68.8% and Anomic: 62.5%), and “hav[ing] a conversation with strangers” (NABW: 71.4% vs. Mild Anomic: 83.3% and Anomic: 60.0%).

**Table 4:** Responses to 5 items administered by the adaptive ACOM related to the BATS story retelling task.

Group	# responses	ACOM_item_content	Not very	Somewhat	Mostly	Completely
NABW	7	have a conversation with strangers	42.9%	28.6%	28.6%	0%
	11	tell a story	9.1%	54.5%	36.4%	0%
	9	tell a joke*	0.0%	55.6%	33.3%	0%
	15	keep a conversation going	13.3%	20.0%	53.3%	13.3%
	11	talk to your closest family member or friend	0.0%	9.1%	27.3%	63.6%
Mild Anomic	12	have a conversation with strangers	25.0%	58.3%	16.7%	0%
	16	tell a story	25.0%	43.8%	25.0%	6.3%
	10	tell a joke	20%	40%	40%	0%
	16	keep a conversation going	6.3%	62.5%	25.0%	6.3%
	9	talk to your closest family member or friend	0.0%	0.0%	66.7%	33.3%
Anomic	15	have a conversation with strangers	13.3%	46.7%	33.3%	6.7%
	16	tell a story	25.0%	37.5%	37.5%	0.0%
	9	tell a joke	22.2%	44.4%	11.1%	22.2%
	16	keep a conversation going	25%	50%	25%	0%
	5	talk to your closest family member or friend	0%	0%	60%	40%

ACOM, Aphasia Communication Outcome Measure (Hula et al., 2015); BATS, Brief Assessment of Transactional Success (Kurland et al., 2021); NABW, Not aphasic by WAB; *One respondent in NABW group answered, “does not apply to me” in response to the item, “How effectively do you tell a joke?”.

## Discussion

There is a growing awareness that there exists a subset of post-stroke survivors who recover enough speech and language ability to perform “within normal limits” on standardized tests of aphasia (e.g., WAB-R; Kertesz, 2007). While they may appear “normal” on tests that are insensitive to mild language impairments, individuals who score NABW on the WAB, effectively a new classification of mild aphasia, are painfully aware of their communication deficits and how those deficits influence their daily activity and participation limitations (Stark et al., 2024). They often cannot access services for aphasia rehabilitation or disability benefits simply because they score above the cutoff for aphasia on a test that is insensitive to mild aphasia (Fromm et al., 2017). Even if they manage to qualify for aphasia intervention, there are no valid, reliable, clinically feasible tools for assessing communication treatment outcomes in mild aphasia. The Scenario Test (van der Meulen et al., 2010), which was recently included as a core communication outcome measure in aphasia (Wallace et al., 2023), unfortunately has ceiling effects for people with mild aphasia.

In the current study, we have demonstrated that the BATS is a tool that discriminates individuals with subclinical aphasia from actual non-aphasic study participants. In comparisons of main concepts produced during story retells between groups of participants with aphasia, their non-aphasic conversation partners, and a non-aphasic control group, conversation partners of participants deemed NABW produced significantly fewer main concepts than the control group (NABW: 57%; NCs: 71%, *p =* 0.0092). Moreover, although the differences did not reach significance in these relatively small groups, there was a clear pattern of non-monotonic declines in performance between consecutive levels of groups (controls > NABW > Mild Anomic > Anomic).

It is also noteworthy that all participants with aphasia, including the subclinical group, appeared “less impaired” on traditional AphasiaBank discourse measures such as picture series descriptions and a procedural discourse task, as compared to their BATS story retells. This finding was not surprising, given the increased task difficulty inherent in the BATS story retell tool. Unlike traditional picture description tasks for acquiring connected discourse in aphasia, the BATS requires story retelling without picture support. This increases task demand, such as reliance on attention, memory, and executive functions. For example, to be successful, the story reteller must focus on fleeting visual and/or auditory information, remember both story gist and details, and produce a coherently sequenced narrative structure, all without current visual cues. This increased cognitive load, absent when a picture is available for story reference, may explain in part why all participants with aphasia conveyed higher percentages of main concepts for traditional stimuli than for BATS stimuli.

It has been proposed that a plausible driver of persistent communication deficits in mild and subclinical aphasia may be impairment of cognitive nonlinguistic mechanisms such as attention (Murray, 2012; Murray et al., 1998, 2006), verbal short-term and working memory (Silkes et al., 2021), conceptual short-term memory (Salis et al., 2021), and executive functions (Frankel et al., 2007; Ramsberger, 2005). These psychological mechanisms, working in concert with linguistic processes, are not as heavily drawn upon during traditional picture description tasks. They are, however, fundamental to supporting real-world communication tasks such as the BATS story retell, in which dyads use joint action to co-construct the stories. In their seminal study of co-constructed story retelling, Ramsberger and Rende (2002) demonstrated that language deficits were only moderately related to conversational success in persons with aphasia. Ramsberger (2005) later suggested that attention and executive functions may have contributed, in addition to language ability.

Both attention (e.g., Hula and McNeil, 2008) and working memory (Baddeley and Hitch, 1974; Baddeley, 2003) have long been acknowledged to involve limited-capacity processing resources that are flexibly allocated to meet task demands. The idea has also been advanced that executive/attentional deficits that impair goal-directed behavior can account for macrolevel discourse impairments such as failing to activate and sustain a cohesive, coherent narrative (Alexander, 2006). In their clinical model of executive functions, Solhberg and Mateer (2001) note how multiple domains of their model apply to conversation. They can account for impaired communication, including initiation and drive, response inhibition, task persistence, organization, generative thinking, and self-awareness.

While each of these examples describes plausible mechanisms for disturbing different processes that underpin successful communication, they are mostly described from a speaker-centric perspective. Importantly, whereas cognitive non-linguistic mechanisms contribute to transactional success in monologic discourse, such as picture description, they become even more critical in real-world communication tasks such as story retelling. In this communicative interactional context, where a story may be jointly produced, memory, attention, and executive function support verbal and nonverbal linguistic mechanisms in the dynamic co-construction of the story retell. As Carragher et al. (2023) observed, co-constructed communication is its own discourse genre, “…a type of semi-structured dialogue … [which] in comparison to more structured discourse genres…, involves increased availability of context, interaction with a communication partner and options for multi-modal communication” (p. 4). These added steps in complexity also increase task demands, bringing co-constructed communication closer to everyday language use, for example, in conversation.

The story retell analysis examines the current investigation’s focus on monologic story retells before and after dyadic co-constructed communication. Thus, while opportunities for multimodal communication *supported* the co-construction of meaning during the topic-constrained conversations, the focus of the current study is on meaning conveyed through monologic spoken language by persons with aphasia and their conversation partners. The story retellings produced by the partner were products of the complex, interactional, multi-modal processes of establishing common ground as they jointly spoke about and understood each story. These fundamental aspects of situated language use (Clark, 1996; Doedens and Meteyard, 2020) provide a means of ensuring mutual understanding, from which we can derive ecologically valid evidence of communication success in aphasia.

It is in this arena that the group of participants with subclinical aphasia expresses their chief complaint of chronic aphasia despite testing within normal limits on language tasks that are devoid of interactional context. When asked to reflect on how effectively they perform in a variety of real-world communication scenarios, the higher the task demand, the more closely the self-perception of participants deemed NABW aligned with that of other participants with aphasia. For example, nearly two-thirds responded “completely” to (*How effectively do you…*) *…talk to your closest family member or friend.* In comparison, nearly three-fourths responded “not very” or “somewhat” to the item, *…have a conversation with strangers.* The NABW group was an outlier compared to the Mild Anomic and Anomic groups; the latter two groups predominantly responded “mostly” to the less demanding conversation task with familiar partners. But all three groups were less confident in their effectiveness when talking with strangers. Similarly, the NABW showed more confidence than the other two groups on the item, …*keep a conversation going*, while all three groups showed less confidence in the more demanding items, …*tell a joke* and …*tell a story.*

In retrospect, it would have been ideal to have ACOM self-ratings from all the participants with aphasia on all the items that most closely aligned with the BATS story retelling co-constructed communication task, including … *correct mistakes you make when you talk, … follow a story someone tells, … talk about movies that you have seen, … find the words you want to say during conversation, … follow conversation about familiar topics,* etc. Unfortunately, given the limitations of the adaptive shortened version of the ACOM, we were unable to control for item presentation, and many of the most relevant items were not presented to a high enough number of participants. Nonetheless, with just a snapshot of self-ratings from enough participants on a handful of story-retelling relevant items, it is clear that self-perception of communication deficits is more closely aligned with partner retell performance on the BATS than what is captured by traditional monologic discourse tasks or standardized measures of language, especially for people deemed NABW.

That vast gap between the ‘subclinical’ diagnostic label and the everyday functional communicative deficits experienced by individuals who know with certainty that they are not ‘normal’ should provide an impetus to interested parties to do things differently. As Fromm et al. (2017) note, in response to these individuals’ frustration regarding the chasm between the label and their lived experience, “clinicians and families should take those comments quite seriously and validate such concerns” (p. 767). At the very least, when it comes to testing individuals with mild aphasia, we ought to stop using measurement tools that are insensitive to the full range of aphasia severity.

The current study lends support for including the BATS as a measure of functional communication in the core outcome set of measures used in aphasia research and intervention. Unlike traditional monologic measures of connected speech, which may discriminate between non-aphasic and subclinical populations but do not reflect real-world communication, the BATS protocol is designed to acquire a story retell from a non-aphasic conversation partner following co-construction of the story with a participant with aphasia. This activity mirrors real-world functional communication. Furthermore, unlike The Scenario Test (TST; van der Meulen et al., 2010), which was recently included as a core outcome measurement instrument for communication (ROMA-2; Wallace et al., 2023), the BATS is not prone to ceiling effects for individuals with mild-to-moderate aphasia. As we continue to develop methods to improve the tool, including leveraging large language models to (1) automate the analysis of story retells and (2) generate main concepts for novel stories, we hope to demonstrate the feasibility and appropriateness of the BATS tool for measuring communication deficits, even the so-called ‘mild’ persistent deficits observed in people with subclinical aphasia.

## Data Availability

Publicly available datasets were analyzed in this study. This data can be found here: AphasiaBank.

## Footnotes

1
https://www.assemblyai.com/products/speech-to-text
